# Net clinical benefit analysis of non-vitamin K antagonist oral anticoagulants in patients with atrial fibrillation and chronic kidney disease: Protocol for a systematic review and meta-analysis

**DOI:** 10.1097/MD.0000000000016194

**Published:** 2019-06-28

**Authors:** Nan-Nan Shen, Xue-Min Zhang, Ke-Jia Le, An-Hua Wei, Yue Wu, Zhi-Chun Gu

**Affiliations:** a Department of Pharmacy, Affiliated Hospital of Shaoxing University, Shao Xing, Zhejiang Province; b Dali Prefecture Hospital of Traditional Chinese Medicine, Da Li, Yunnan Province; c Department of Pharmacy, Renji Hospital, School of Medicine, Shanghai Jiaotong University, Shanghai; d Department of Pharmacy, Tongji Hospital, Tongji Medical College, Huazhong University of Science and Technology; e Department of Pharmacy, Wuhan University, Renmin Hospital, Wuhan, China.

**Keywords:** atrial fibrillation, chronic kidney disease, net clinical benefit, non-vitamin K antagonist oral anticoagulants

## Abstract

**Background:**

Atrial fibrillation (AF) is increasingly prevalent in chronic kidney disease (CKD) patients. The efficacy and safety of non-vitamin K antagonist oral anticoagulants (NOACs) in AF and CKD patients remains unknown. This systematic review and meta-analysis will mainly assess net clinical benefit (NCB) property of NOACs versus warfarin in patients with AF and CKD by a pooled-analysis.

**Methods:**

We will search Medline, Embase, Cochrane Library, and Clinical Trials.gov Website comprehensively for eligible randomized controlled trials that report the efficacy and safety outcomes according to renal function of NOACs. Relative risks and their 95% confidence intervals will be calculated using fixed- and random-effects models. Subgroup, sensitivity, and regression analyses will be performed to evaluate intertrial heterogeneity and bias of the results. NCB that balance stroke/systemic embolism (SSE) and major bleeding will be calculated using Singer's method.

**Results:**

This systemic review and meta-analysis will evaluate the NCB of NOACs versus warfarin via SSE, major bleeding and all-cause death in patients with CKD.

**Conclusions:**

This study will provide new evidence for clinical profile of NOACs on SSE, major bleeding, all-cause death, and NCB in CKD patients.

**PROSPERO registration number:**

CRD42019116940.

## 1 Introduction

The prevalence of atrial fibrillation (AF) is increasingly higher in chronic kidney disease (CKD) patients.^[1]^ Of note, both AF and CKD increase the risk of stroke and systemic thromboembolism.^[2]^ CKD is associated with an enhanced risk of cardiovascular disease, and it is an independent risk factor of thromboembolism and bleeding.^[3]^ In addition, CKD is widely considered as a predictor for the low time in therapeutic range (TTR) and superimposed platelet dysfunction in AF patients treated with warfarin.^[4]^ Thus, patients with concomitant AF and CKD are at a higher risk of stroke or embolism as well as bleeding.

Stroke/systemic embolism (SSE) is a leading cause of mortality and morbidity, non-vitamin K antagonist oral anticoagulants (NOACs), due to their favorable profile of efficacy and safety, represents an optimal therapeutic option in AF.^[5]^ In recent years, NOACs account for most of oral anticoagulants prescribed for patients with newly diagnosed AF.^[6]^ Meanwhile, the use of NOACs in CKD is also increasing in spite of the limited evidence on these fragile patients.^[7]^ It is acknowledged that renal clearance of NOACs are dabigatran 80%, edoxaban 50%, rivaroxaban 36%, apixaban 27%, respectively.^[8]^ Therefore, the accumulation of free blood concentration may lead to a subsequent bleeding risk in CKD patients.^[9]^ Previous studies only evaluated the efficacy and safety of individual NOACs on thrombotic and bleeding risk in general AF patients.^[10,11]^ There are no head-to-head risk comparisons among the NOACs in CKD patients. Currently, the direct data on NOACs from randomized clinical trials (RCTs) and real word studies in patients with concomitant AF and CKD are limited. Only a few studies assessed the efficacy and safety of individual NOACs across CKD subgroup analysis.^[12,13]^ Compared with warfarin, NOACs have been proved to reduce SSE and major bleeding in CKD patients, but number of patients needed to treat (NNT) and net clinical benefit (NCB) has not been explored.^[14,15]^ In recent years, the NCB property of NOACs has been developed and gradually to balance the comprehensive risk of SSE and bleeding in AF.^[16]^ Noteworthy, Pelliccia et al reported that apixaban and dabigatran were superior to warfarin in terms of the NCB.^[17]^

Current evidence on NCB of AF is derived mainly from observational studies and registry-based cohorts, data from RCTs, especially for patients with AF and CKD is quite limited.^[18–20]^ Hence, this systematic review and meta-analysis will mainly assess the NCB property of NOACs in patients with concomitant AF and CKD by a pooled analysis and available findings might provide a viable option of NOACs in patients with AF and CKD.

## 2 Methods

### 2.1 Data sources and search strategy

The study will be conducted in accordance with the standards of the preferred reporting items for systematic reviews and meta-analyses statement. Medline, Embase, and Cochrane Library will be comprehensively searched to identify all potentially eligible studies. The search strategy will be conducted as follows: we will include the following terms for the theme “NOACs:” “Pradaxa” or “dabigatran” or “Xarelto” or “rivaroxaban” or “Eliquis” or “apixaban” or “Savaysa” or “edoxaban” or “Bevyxxa” or “betrixaban” or “Non-vitamin K antagonist oral anticoagulants” or “direct oral anticoagulants” or “NOACs” or “DOACs” or “novel oral anticoagulants” or “new oral anticoagulants” or “factor Xa inhibitors” or “factor IIa inhibitors.” For the theme “atrial fibrillation,” we will use “atrial fibrillation” or “AF.” For the theme “RCTs,” we will include the following terms: “randomized controlled trial” or “controlled clinical trial” or “clinical trial.” Then we will use the Boolean operator “AND” to combine the 3 comprehensive search themes. In addition, unpublished trials will be identified from the ClinicalTrials.gov Website.

### 2.2 Study selection

Studies will be selected if they met the following inclusion and exclusion criteria:

(1) study involving phase III RCTs of patients with AF and receiving one of the NOACs as compared to warfarin;
(2) detail information for renal function and related outcomes of patients are reported in study;
(3) RCTs that include patients with mitral stenosis or prosthetic cardiac valves, mean or median follow-up <6 months, <200 subjects, and NOAC phase II will be excluded. For trials reporting multiple publications, the most relevant data will be extracted.

### 2.3 Study outcomes

Pre-specified outcomes are SSE, major bleeding, all-cause death, and NCB. Major bleeding is defined according to the International Society of Thrombosis and Hemostasis. The data analysis will follow the intention-to-treat principle, creatinine clearance (CrCl) represents renal function following the Cockcroft-Gault formula, and classified as no CKD group (CrCl >80 ml/min), mild CKD group (CrCl = 50–80 ml/min), and moderate CKD group (CrCl 30–50 ml/min).

### 2.4 Data extraction and quality evaluation, and bias assessment

Data will be extracted, including year of publication, duration of follow up, number of patients, mean age, sex, AF type, mean CHADS_2_ score, risk factors, and prior medicine use. The methodological quality of trials will be assessed according to the Cochrane Collaboration Risk of Bias Tool, which focus on random sequence generation, allocation concealment, blinding, incomplete outcome data, selective reporting, and other biases.^[21]^ Funnel plots will be generated to assess for publication bias.^[21]^

### 2.5 Data analysis

Relative risks and 95% confidence intervals of the outcomes in NOACs versus warfarin will be calculated using fixed- and random-effects models. Statistical heterogeneity will be assessed with *I*^2^ test. *I*^2^ of >50% indicate considerable heterogeneity, and *P* value of <0.05 represents a significant heterogeneity.^[22]^ Subgroup analyses will be conducted following renal function group. The number of patients NNT to prevent 1 event will be calculated as: (1/absolute risk reduction) × 100, where absolute risk reduction will be rate difference (event rates per 100 patients-year on warfarin minus event rates per 100 patients-year on NOACs).^[18]^ The NCB of NOACs compared with warfarin will be calculated using the follow formula: (rate of SSE on warfarin minus the rate of SSE on NOACs) − weight × (rate of major bleeding on NOACs minus rate of major bleeding on warfarin), where rate is event rates per 100 patients-year. We will assign their weighting factor of 1.5, and also provide additional sensitivity analysis using weighted factor of 1.0 and 2.0.^[16]^ Meta-regression analysis will be performed to explore the influence of these factors on outcomes. Sensitivity analysis will be conducted to evaluate the robustness of the results. In addition, interaction analysis will be applied for detecting the treatment discrepancies among different renal function groups. All statistical analyses will be performed using STATA software (version13, Statacorp, College Station, TX), and *P* < .05 indicate a statistically significant difference.

## 3 Discussion

CKD is associated with high risk of both stroke and major bleeding, and the assessment of balance between the risk of thromboembolic and bleeding events is essential in AF patients with CKD^2^. Many CKD patients receive inadequate oral anticoagulation (OAC) in clinical setting due to worrying about bleeding while on OAC. Therefore, it is necessary to provide suitable anticoagulant therapy for AF patients companied with CKD. The updated clinical guidelines on the management of AF recommend NOACs, due to stable safety and efficacy, which is favor for stroke prevention over warfarin in general NVAF patients.^[23]^ However, there is no definite recommendation about the choice of warfarin or NOACs for stroke prevention in patients with CKD. Furthermore, the lack evidence for NCB property of NOACs versus warfarin for AF patients with varying degrees of renal function, leading to clinicians difficult to choose suitable OAC for these patients. For this reason, we will assess the efficacy, safety, and NCB of NOACs in AF patients with different stages of renal function.

Although CKD is not regarded as component factor of CHA_2_DS_2_-VASc score, it is closely associated with its risk factors, such as congestive heart failure, hypertension, age, diabetes, and it is considered a risk factor for predicting the bleeding risk in HAS-BLED score.^[24]^ It is recognized that CKD is related to a suboptimal TTR in AF patients received warfarin therapy.^[4]^ CKD might contribute to poor control of TTR via increasing risk of thromboembolism and bleeding.^[25]^ In sub-analysis of previous studies, NOACs reduced the risk of SSE and major bleeding events in CKD patients.^[26–29]^ For this issue, we will integrate included studies for powerful statistics to estimate NCB that incorporates the risk for SSE and major bleeding events of CKD patients receiving NOACs, and provide a greater NCB basis for the option on optimal anticoagulant therapy in AF patients.

Several limitations might be worth addressed in this study. First, the included trials may not be directly designed to evaluate the efficacy and safety of NOACs in patients with CKD. The differences in patient demographics, bleeding risk factors, concomitant drugs may be unsolved for further analysis. However, meta-regression analysis will be performed to assess available potential effect in baseline characteristics. Second, the statistical Singer's method using 1.5 weighted index may not account for all clinical variables.^[16]^ Thus, sensitivity analysis will be conducted using weighted factor of 1.0 and 2.0. Finally, the absence of head-to-head comparisons of NOACs in CKD patients, and limited number of included studies, may lead to an incomprehensive explanation, the result in this study may be applied only to limited scope.

## 4 Conclusions

The results will provide novel evidence for NOACs profile on efficacy, safety, and NCB compared to warfarin in CKD patients.

## Author contributions

Conception and design: Zhi-Chun Gu and Nan-Nan Shen.

Administrative support: Zhi-Chun Gu, An-Hua Wei, and Yue Wu.

Provision of study materials or patients: Nan-Nan Shen and Xue-Min Zhang.

Collection and assembly of data: Nan-Nan Shen, Xue-Min Zhang, and Ke-Jia Le.

Data analysis and interpretation: Zhi-Chun Gu and Nan-Nan Shen.

Manuscript writing: All authors.

Final approval of manuscript: All authors.

**Conceptualization:** Nan-Nan Shen, Xue-Min Zhang, Ke-Jia Le, An-Hua Wei, Yue Wu, Zhichun Gu.

## References

[1] Guo Y Gao J Ye P Comparison of atrial fibrillation in CKD and non-CKD populations: a cross-sectional analysis from the Kailuan study. Int J Cardiol 2019;277:125–9. 30473335 10.1016/j.ijcard.2018.11.098

[2] Olesen JB Lip GY Kamper AL Stroke and bleeding in atrial fibrillation with chronic kidney disease. N Engl J Med 2012;367:625–35. 22894575 10.1056/NEJMoa1105594

[3] Alonso A Lopez FL Matsushita K Chronic kidney disease is associated with the incidence of atrial fibrillation: the Atherosclerosis Risk in Communities (ARIC) study. Circulation 2011;123:2946–53. 21646496 10.1161/CIRCULATIONAHA.111.020982 PMC3139978

[4] Chan KE Giugliano RP Patel MR Nonvitamin K anticoagulant agents in patients with advanced chronic kidney disease or on dialysis with AF. J Am Coll Cardiol 2016;67:2888–99. 27311528 10.1016/j.jacc.2016.02.082

[5] Gu ZC Zhou LY Shen L Non-vitamin K antagonist oral anticoagulants vs. warfarin at risk of fractures: a systematic review and meta-analysis of randomized controlled trials. Front Pharmacol 2018;9:348. 29692734 10.3389/fphar.2018.00348 PMC5903161

[6] Chao TF Chiang CE Lin YJ Evolving changes of the use of oral anticoagulants and outcomes in patients with newly diagnosed atrial fibrillation in Taiwan. Circulation 2018;138:1485–7. 30354355 10.1161/CIRCULATIONAHA.118.036046

[7] Desai NR Krumme AA Schneeweiss S Patterns of initiation of oral anticoagulants in patients with atrial fibrillation-quality and cost implications. Am J Med 2014;127:1075–82. 24859719 10.1016/j.amjmed.2014.05.013

[8] Pelliccia F Rosanio S Marazzi G Efficacy and safety of novel anticoagulants versus vitamin K antagonists in patients with mild and moderate to severe renal insufficiency: focus on apixaban. Int J Cardiol 2016;255:77–81. 10.1016/j.ijcard.2016.09.129 27716554

[9] McCullough PABT Cox KM Assar MD Use of oral anticoagulation in the management of atrial fibrillation in patients with ESRD: pro. Clin J Am Soc Nephrol 2016;11:2079–84. 27797888 10.2215/CJN.02680316 PMC5108189

[10] Tereshchenko LG Henrikson CA Cigarroa J Comparative effectiveness of interventions for stroke prevention in atrial fibrillation: a network meta-analysis. J Am Heart Assoc 2016;5:e003206. 27207998 10.1161/JAHA.116.003206 PMC4889191

[11] Sahay S Nombela-Franco L Rodes-Cabau J Efficacy and safety of left atrial appendage closure versus medical treatment in atrial fibrillation: a network meta-analysis from randomised trials. Heart 2017;103:139–47. 27587437 10.1136/heartjnl-2016-309782

[12] Nielsen PBLD Rasmussen LH Lip GY Renal function and non-vitamin K oral anticoagulants in comparison with warfarin on safety and efficacy outcomes in atrial fibrillation patients: a systemic review and meta-regression analysis. Clin Res Cardiol 2015;104:418–29. 25416564 10.1007/s00392-014-0797-9

[13] Andò GCP Non-vitamin K antagonist oral anticoagulants in atrial fibrillation patients with chronic kidney disease: a systematic review and network meta-analysis. Int J Cardiol 2017;231:162–9. 28007305 10.1016/j.ijcard.2016.11.303

[14] Sardar PCS Herzog E Nairooz R Novel oral anticoagulants in patients with renal insufficiency: a meta-analysis of randomized trials. Can J Cardiol 2014;30:888–97. 25064581 10.1016/j.cjca.2014.04.015

[15] Del-Carpio Munoz F Gharacholou SM Munger TM Meta-analysis of renal function on the safety and efficacy of novel oral anticoagulants for atrial fibrillation. Am J Cardiol 2016;117:69–75. 26698882 10.1016/j.amjcard.2015.09.046

[16] Singer DE Chang Y Fang MC The net clinical benefit of warfarin anticoagulation in atrial fibrillation. Ann Intern Med 2009;151:297–305. 19721017 10.7326/0003-4819-151-5-200909010-00003 PMC2777526

[17] Pelliccia F Rosanio S Marazzi G Efficacy and safety of novel anticoagulants versus vitamin K antagonists in patients with mild and moderate to severe renal insufficiency: focus on apixaban. Int J Cardiol 2016;225:77–81. 27716554 10.1016/j.ijcard.2016.09.129

[18] Watanabe E Yamamoto M Kodama I Net clinical benefit of adding aspirin to warfarin in patients with atrial fibrillation: insights from the J-RHYTHM Registry. Int J Cardiol 2016;212:311–7. 27057949 10.1016/j.ijcard.2016.03.008

[19] Chan PH Huang D Lau CP Net clinical benefit of dabigatran over warfarin in patients with atrial fibrillation stratified by CHA2DS2-VASc and time in therapeutic range. Can J Cardiol 2016;32:1247 doi: 10.1016/j.cjca.2016.01.016. 10.1016/j.cjca.2016.01.016 27118057

[20] Bonde AN Lip GY Kamper AL Net clinical benefit of antithrombotic therapy in patients with atrial fibrillation and chronic kidney disease: a nationwide observational cohort study. J Am Coll Cardiol 2014;64:2471–82. 25500231 10.1016/j.jacc.2014.09.051

[21] Yan YD Zhang C Shen L Net clinical benefit of non-vitamin K antagonist oral anticoagulants for venous thromboembolism prophylaxis in patients with cancer: a systematic review and trade-off analysis from 9 randomized controlled trials. Front Pharmacol 2018;9:575 doi: 10.3389/fphar.2018.00575. 29946255 10.3389/fphar.2018.00575 PMC6005885

[22] Wei AH Gu ZC Zhang C Increased risk of myocardial infarction with dabigatran etexilate: fact or fiction? A critical meta-analysis of over 580,000 patients from integrating randomized controlled trials and real-world studies. Int J Cardiol 2018;267:1–7. 29801762 10.1016/j.ijcard.2018.05.048

[23] January CT Wann LS Calkins H 2019 AHA/ACC/HRS focused update of the 2014 AHA/ACC/HRS guideline for the management of patients with atrial fibrillation: a report of the American College of Cardiology/American Heart Association task force on clinical practice guidelines and the heart rhythm society. Heart Rhythm 2019;pii: S1547-5271(19)30037-2. doi: 10.1016/j.hrthm.2019.01.024. 10.1016/j.hrthm.2019.01.024 30703530

[24] Uchiyama S Hori M Matsumoto M Net clinical benefit of rivaroxaban versus warfarin in Japanese patients with nonvalvular atrial fibrillation: a subgroup analysis of J-ROCKET AF. J Stroke Cerebrovasc Dis 2014;23:1142–7. 24189454 10.1016/j.jstrokecerebrovasdis.2013.10.001

[25] Apostolakis SSR Olshansky B Lip GYH Factors affecting quality of anticoagulation control among patients with atrial fibrillation on warfarin: the SAMe-TT(2)R(2) score. Chest 2013;144:1555–63. 23669885 10.1378/chest.13-0054

[26] Hijazi Z Hohnloser SH Oldgren J Efficacy and safety of dabigatran compared with warfarin in relation to baseline renal function in patients with atrial fibrillation: a RE-LY (randomized evaluation of long-term anticoagulation therapy) trial analysis. Circulation 2014;129:961–70. 24323795 10.1161/CIRCULATIONAHA.113.003628

[27] Fox KA Piccini JP Wojdyla D Prevention of stroke and systemic embolism with rivaroxaban compared with warfarin in patients with non-valvular atrial fibrillation and moderate renal impairment. Eur Heart J 2011;32:2387–94. 21873708 10.1093/eurheartj/ehr342

[28] Hohnloser SH Hijazi Z Thomas L Efficacy of apixaban when compared with warfarin in relation to renal function in patients with atrial fibrillation: insights from the ARISTOTLE trial. Eur Heart J 2012;33:2821–30. 22933567 10.1093/eurheartj/ehs274

[29] Bohula EA Giugliano RP Ruff CT Impact of renal function on outcomes with edoxaban in the ENGAGE AF-TIMI 48 trial. Circulation 2016;134:24–36. 27358434 10.1161/CIRCULATIONAHA.116.022361

